# Supplementary material for: Genetic Variants at the APOE Locus Predict Cardiometabolic Traits and Metabolic Syndrome: A Taiwan Biobank Study
Source: Genes (Basel). 2022 Jul 29;13(8):1366. doi: 10.3390/genes13081366 (PMC9407549; doi:10.3390/genes13081366)
Supplement: Supplementary file 1 [file genes-13-01366-s001.zip › genes-1821435-supplementary.pdf]

Supplementary Method 1

Definitions of hypertension, diabetes mellitus, current smoking, microalbuminuria and metabolic syndrome

Hypertension was defined as systolic blood pressure (BP) of ≥140 mmHg, diastolic BP of ≥90 mmHg or a self-reported history of hypertension. Diabetes mellitus (DM) was defined as a fasting plasma glucose level of ≥126 mg/dL, a glycohemoglobin value of ≥6.5%, or a self-reported history of DM. Current smoking was defined as regular cigarette smoking at the time of survey. Because of the absence of urine creatinine level, only spot urine albumin level was used for the evaluation of urine albumin. Microalbuminuria was defined as urine albumin of ≥30 mg/L.

Because medication histories were unavailable, metabolic syndrome characteristics were based on the recent update of the third report of the National Cholesterol Education Program's Adult Treatment Panel III criteria [1] with modifications. Participants with three or more of the following attributes are typically defined as having metabolic syndrome: (1) BP of ≥130/85 mmHg or a history of hypertension; (2) triglyceride level of ≥150 mg/dL; (3) high-density lipoprotein cholesterol level of <40 mg/dL for men or <50 mg/dL for women; (4) fasting plasma glucose of ≥100 mg/dL or a history of DM; and (5) waist circumference of >90 cm for men or>80 cm for women.

Supplementary Table S1. Lead single-nucleotide polymorphisms for serum lipid levels around the *APOE* gene region in regional association studies with conditional analysis.

SNP	Position (GRCh37)	Gene	Ref/Alt	MAF	HWE	Exon number	Exonic Function	mRNA sequence	Amino acid Change
rs429358	45411941	*APOE*	T/C	0.0865	0.3318	4	nonsynonymous SNV	c.T466C	p.C156R
rs7412	45412079	*APOE*	C/T	0.0737	0.2991	4	nonsynonymous SNV	c.C604T	p.R202C
rs439401	45414451	*APOE; APOC1*	T/C	0.4063	0.7327	intergenic	--	--	--
rs438811	45416741	*APOC1*	C/T	0.1753	0.4214	upstream	--	--	--
rs11672748	45490192	*CLPTM1*	A/G	0.4856	0.0732	intron	--	--	--
rs3786505	45490570	*CLPTM1*	A/G	0.4855	0.0883	10	synonymous SNV	c.A927C	p.Pro309=

SNP: single nucleotide polymorphism, Ref/Alt: reference/alternate allele, HWE: Hardy-Weinberg equilibrium, MAF: minor allele frequency, mRNA: messenger RNA.

Supplementary Table S2. Association of the *APOE* rs7412 genotype with metabolic and hematological phenotypes

Clinical and laboratory parameters	Beta	SE	*p* value
Anthropology			
Age (years)	-0.0539	0.1014	0.5953
Waist circumference (cm)	0.0048	0.0484	0.9203
Waist-hip ratio	-0.0005	0.0005	0.3154
Body mass index (kg/m^2)	0.0925	0.0345	0.0074
Blood Pressure			
Systolic BP† (mmHg)	-0.3239	0.1454	0.0260
Diastolic BP† (mmHg)	-0.0802	0.0948	0.3976
Mean BP† (mmHg)	-0.1614	0.1042	0.1214
Lipid profiles			
Total cholesterol# (mg/dL)	-0.0189	0.0006	3.24×10^{-193}
HDL-cholesterol# (mg/dL)	0.0234	0.0010	2.14×10^{-113}
LDL-cholesterol# (mg/dL)	-0.0759	0.0010	$<10^{-307}$
Triglyceride# (mg/dL)	0.0248	0.0021	1.18×10^{-32}
Glucose metabolism			
Fasting plasma glucose†† (mg/dL)	-0.2058	0.1436	0.1520
HbA1C†† (%)	-0.0065	0.0057	0.2567
Uric acid			
Uric acid††† (mg/dL)	-0.0280	0.0106	0.0082
Renal function			
Creatinine (mg/dL)	0.0032	0.0026	0.2046
eGFR (mL/min/1.73 m^2)	-0.4918	0.2060	0.0170
Albuminuria (mg/L)	-0.0056	0.0043	0.1933
Liver function			
AST (U/L)	0.1510	0.1151	0.1894
ALT (U/L)	0.0517	0.1778	0.7714
γGT (U/L)	0.2473	0.2973	0.4055
Serum albumin (g/dL)	-0.0094	0.0021	8.67×10^{-6}
Total bilirubin (mg/dL)	-0.0042	0.0025	0.1010
Hematological parameters			
Leukocyte count (10^3/μL)	-0.0069	0.0146	0.6393
Hematocrit (%)	0.0103	0.0332	0.7570
Platelet count (10^3/μL)	0.2847	0.5420	0.5994
Red blood cell count (10^6/μL)	-0.0077	0.0042	0.0675
Hemoglobin (g/dL)	0.0135	0.0117	0.2500
Atherosclerotic risk factors			
Diabetes mellitus (%)	-0.0596	0.0347	0.0859
Hypertension (%)	-0.0371	0.0254	0.1442
Current smoking (%)	0.0199	0.0345	0.5644
Gout (%)	-0.0440	0.0514	0.3921
Microalbuminuria (%)	-0.0103	0.0304	0.7339
Metabolic syndrome (%)	0.0372	0.0261	0.1539

P: adjusted for age, sex, BMI, and current smoking; age: adjusted for sex, BMI and current smoking; BMI: adjusted for age, sex and smoking; current smoking: adjusted for age, sex and BMI.
Participants were analyzed after the exclusion of those with a history of †hypertension, ††diabetes mellitus, †††gout, and #hyperlipidemia.

Abbreviations: SE, standard error; BP, blood pressure; HDL, high-density lipoprotein; LDL, low-density lipoprotein; HbA1C, hemoglobin A1C; eGFR, estimated glomerular filtration rate; BUN, blood urea nitrogen; AST, aspartate aminotransferase; ALT, alanine aminotransferase; γGT, γ-Glutamyl transferase; BMI, body mass index.

Supplementary Table S3. Association of the *APOE* rs429358 genotype with metabolic and hematological phenotypes

Clinical and laboratory parameters	Beta	SE	*p* value
Anthropology			
Age (years)	0.0780	0.0941	0.4071
Waist circumference (cm)	-0.0250	0.0449	0.5783
Waist-hip ratio	-0.0014	0.0005	0.0034
Body mass index (kg/m^2)	-0.0362	0.0321	0.2589
Blood Pressure			
Systolic BP† (mmHg)	0.0913	0.1352	0.4997
Diastolic BP† (mmHg)	-0.0621	0.0882	0.4815
Mean BP† (mmHg)	-0.0110	0.0969	0.9100
Lipid profiles			
Total cholesterol# (mg/dL)	0.0053	0.0007	2.35×10^{-14}
HDL-cholesterol# (mg/dL)	-0.0136	0.0011	9.07×10^{-34}
LDL-cholesterol# (mg/dL)	0.0198	0.0012	6.05×10^{-62}
Triglyceride# (mg/dL)	0.0166	0.0023	2.15×10^{-13}
Glucose metabolism			
Fasting plasma glucose†† (mg/dL)	-0.1449	0.1332	0.2767
HbA1C†† (%)	-0.0085	0.0053	0.1102
Uric acid			
Uric acid††† (mg/dL)	-0.0078	0.0098	0.4304
Renal function			
Creatinine (mg/dL)	-0.0041	0.0024	0.0874
eGFR (mL/min/1.73 m^2)	0.6623	0.1913	0.0005
Albuminuria (mg/L)	-0.0070	0.0040	0.0799
Liver function			
AST (U/L)	-0.2224	0.1067	0.0372
ALT (U/L)	-0.6922	0.1649	2.70×10^{-5}
γGT (U/L)	-0.2135	0.2760	0.4393
Serum albumin (g/dL)	-0.0048	0.0020	0.0150
Total bilirubin (mg/dL)	-0.0024	0.0024	0.3139
Hematological parameters			
Leukocyte count (10^3/μL)	-0.0010	0.0136	0.9419
Hematocrit (%)	-0.0296	0.0308	0.3374
Platelet count (10^3/μL)	-1.5290	0.5030	0.0024
Red blood cell count (10^6/μL)	-0.0010	0.0039	0.7963
Hemoglobin (g/dL)	-0.0103	0.0109	0.3447
Atherosclerotic risk factors			
Diabetes mellitus (%)	-0.0663	0.0325	0.0416
Hypertension (%)	0.0018	0.0236	0.9403
Current smoking (%)	-0.0103	0.0326	0.7519
Gout (%)	0.0420	0.0469	0.3711
Microalbuminuria (%)	-0.0147	0.0285	0.6049
Metabolic syndrome (%)	0.1827	0.0239	2.09×10^{-14}

Abbreviations, adjusted condition and participant enrollment as in Supplementary Table S1.

Supplementary Table S4. Association of the *APOC1* rs438811 genotypes with metabolic and hematological phenotypes

Clinical and laboratory parameters	Beta	SE	*p* value
Anthropology			
Age (years)	0.0295	0.0697	0.6720
Waist circumference (cm)	-0.0192	0.0333	0.5635
Waist-hip ratio	-0.0012	0.0003	0.0005
Body mass index (kg/m^2)	0.0241	0.0237	0.3110
Blood Pressure			
Systolic BP† (mmHg)	-0.0736	0.1001	0.4620
Diastolic BP† (mmHg)	-0.0742	0.0653	0.2553
Mean BP† (mmHg)	-0.0740	0.0717	0.3019
Lipid profiles			
Total cholesterol# (mg/dL)	-0.0082	0.0005	1.63×10^{-56}
HDL-cholesterol# (mg/dL)	0.0002	0.0006	0.6896
LDL-cholesterol# (mg/dL)	-0.0309	0.0008	$<10^{-307}$
Triglyceride# (mg/dL)	0.0283	0.0014	1.61×10^{-85}
Glucose metabolism			
Fasting plasma glucose†† (mg/dL)	-0.1834	0.0988	0.0633
HbA1C†† (%)	-0.0059	0.0039	0.1345
Uric acid			
Uric acid††† (mg/dL)	-0.0191	0.0073	0.0088
Renal function			
Creatinine (mg/dL)	2.10×10^{-5}	0.0018	0.9904
eGFR (mL/min/1.73 m^2)	0.0796	0.1416	0.5740
Albuminuria (mg/L)	-0.0041	0.0030	0.1695
Liver function			
AST (U/L)	-0.1149	0.0792	0.1471
ALT (U/L)	-0.4749	0.1222	0.0001
γGT (U/L)	-0.0871	0.2046	0.6701
Serum albumin (g/dL)	-0.0068	0.0015	2.94×10^{-6}
Total bilirubin (mg/dL)	-0.0036	0.0018	0.0396
Hematological parameters			
Leukocyte count (10^3/μL)	-0.0079	0.0101	0.4327
Hematocrit (%)	-0.0105	0.0228	0.6464
Platelet count (10^3/μL)	-0.7619	0.3726	0.0409
Red blood cell count (10^6/μL)	-0.0046	0.0029	0.1126
Hemoglobin (g/dL)	0.0022	0.0081	0.7880
Atherosclerotic risk factors			
Diabetes mellitus (%)	-0.0571	0.0239	0.0168
Hypertension (%)	-0.0052	0.0175	0.7670
Current smoking (%)	0.0035	0.0240	0.8842
Gout (%)	-0.0118	0.0352	0.7386
Microalbuminuria (%)	-0.0115	0.0210	0.5851
Metabolic syndrome (%)	0.0977	0.0179	4.90×10^{-8}

Abbreviations, adjusted condition and participant enrollment as in Supplementary Table S1.

Supplementary Table S5. Association of the *APOE* rs439401 genotypes with metabolic and hematological phenotypes

Clinical and laboratory parameters	Beta	SE	*p* value
Anthropology			
Age (years)	-0.0307	0.0538	0.5686
Waist circumference (cm)	0.0084	0.0257	0.7428
Waist-hip ratio	-0.0006	0.0003	0.0162
Body mass index (kg/m^2)	0.0359	0.0183	0.0497
Blood Pressure			
Systolic BP† (mmHg)	-0.1381	0.0772	0.0737
Diastolic BP† (mmHg)	-0.1101	0.0503	0.0287
Mean BP† (mmHg)	-0.1194	0.0553	0.0308
Lipid profiles			
Total cholesterol# (mg/dL)	-0.0032	0.0004	2.80×10^{-18}
HDL-cholesterol# (mg/dL)	0.0036	0.0006	1.17×10^{-9}
LDL-cholesterol# (mg/dL)	-0.0151	0.0006	2.82×10^{-124}
Triglyceride# (mg/dL)	0.0156	0.0012	2.57×10^{-38}
Glucose metabolism			
Fasting plasma glucose†† (mg/dL)	-0.0809	0.0763	0.2886
HbA1C†† (%)	-0.0024	0.0030	0.4371
Uric acid			
Uric acid††† (mg/dL)	-0.0083	0.0056	0.1417
Renal function			
Creatinine (mg/dL)	0.0027	0.0014	0.0504
eGFR (mL/min/1.73 m^2)	-0.0010	0.1093	0.9924
Albuminuria (mg/L)	-0.0026	0.0023	0.2588
Liver function			
AST (U/L)	-0.0068	0.0610	0.9114
ALT (U/L)	-0.0896	0.0943	0.3418
γGT (U/L)	0.0119	0.1576	0.9396
Serum albumin (g/dL)	-0.0039	0.0011	0.0005
Total bilirubin (mg/dL)	-0.0036	0.0014	0.0074
Hematological parameters			
Leukocyte count (10^3/μL)	-0.0054	0.0078	0.4899
Hematocrit (%)	-0.0097	0.0176	0.5803
Platelet count (10^3/μL)	0.0183	0.2874	0.9493
Red blood cell count (10^6/μL)	-0.0013	0.0022	0.5607
Hemoglobin (g/dL)	-0.0019	0.0062	0.7642
Atherosclerotic risk factors			
Diabetes mellitus (%)	-0.0351	0.0182	0.0537
Hypertension (%)	-0.0241	0.0134	0.0727
Current smoking (%)	-0.0031	0.0185	0.8651
Gout (%)	0.0267	0.0270	0.3226
Microalbuminuria (%)	0.0044	0.0161	0.7862
Metabolic syndrome (%)	0.0421	0.0139	0.0025

Abbreviations, adjusted condition and participant enrollment as in Supplementary Table S1.

Supplementary Table S6. Association of the *CLPTM1* rs3786505 genotype with metabolic and hematological phenotypes

Clinical and laboratory parameters	Beta	SE	*p* value
Anthropology			
Age (years)	0.0607	0.0529	0.2510
Waist circumference (cm)	0.0067	0.0252	0.7893
Waist-hip ratio	0.0002	0.0003	0.3808
Body mass index (kg/m^2)	-0.0131	0.0180	0.4669
Blood Pressure			
Systolic BP† (mmHg)	0.1287	0.0759	0.0900
Diastolic BP† (mmHg)	0.0912	0.0495	0.0655
Mean BP† (mmHg)	0.1037	0.0544	0.0566
Lipid profiles			
Total cholesterol# (mg/dL)	-0.0009	0.0004	0.0187
HDL-cholesterol# (mg/dL)	0.0038	0.0005	5.99×10^{-16}
LDL-cholesterol# (mg/dL)	-0.0045	0.0006	1.67×10^{-14}
Triglyceride# (mg/dL)	0.0000	0.0011	0.9832
Glucose metabolism			
Fasting plasma glucose†† (mg/dL)	0.1040	0.0751	0.1659
HbA1C†† (%)	0.0001	0.0030	0.9740
Uric acid			
Uric acid††† (mg/dL)	-0.0018	0.0055	0.7426
Renal function			
Creatinine (mg/dL)	0.0005	0.0013	0.7051
eGFR (mL/min/1.73 m^2)	0.0952	0.1074	0.3754
Albuminuria (mg/L)	-0.0004	0.0023	0.8618
Liver function			
AST (U/L)	-0.0083	0.0599	0.8897
ALT (U/L)	-0.0435	0.0926	0.6387
γGT (U/L)	0.1174	0.1549	0.4487
Serum albumin (g/dL)	-0.0011	0.0011	0.3101
Total bilirubin (mg/dL)	-0.0026	0.0013	0.0538
Hematological parameters			
Leukocyte count (10^3/μL)	0.0132	0.0076	0.0823
Hematocrit (%)	-0.0307	0.0173	0.0762
Platelet count (10^3/μL)	0.6830	0.2826	0.0157
Red blood cell count (10^6/μL)	-0.0090	0.0022	4.22×10^{-5}
Hemoglobin (g/dL)	-0.0078	0.0061	0.2010
Atherosclerotic risk factors			
Diabetes mellitus (%)	0.0115	0.0178	0.5173
Hypertension (%)	0.0031	0.0132	0.8132
Current smoking (%)	0.0055	0.0181	0.7637
Gout (%)	0.0191	0.0265	0.4704
Microalbuminuria (%)	-0.0173	0.0159	0.2771
Metabolic syndrome (%)	-0.0209	0.0137	0.1282

Abbreviations, adjusted condition and participant enrollment as in Supplementary Table S1.

Supplementary Table S7. Association of the *CLPTM1* rs11672748 genotype with metabolic and hematological phenotypes

Clinical and laboratory parameters	Beta	SE	*p* value
Anthropology			
Age (years)	0.0596	0.0529	0.2596
Waist circumference (cm)	0.0052	0.0252	0.8369
Waist-hip ratio	0.0002	0.0003	0.4030
Body mass index (kg/m^2)	-0.0122	0.0180	0.4969
Blood Pressure			
Systolic BP† (mmHg)	0.1301	0.0759	0.0867
Diastolic BP† (mmHg)	0.0922	0.0495	0.0625
Mean BP† (mmHg)	0.1049	0.0544	0.0540
Lipid profiles			
Total cholesterol# (mg/dL)	-0.0003	0.0004	0.3958
HDL-cholesterol# (mg/dL)	0.0027	0.0006	3.96×10^{-6}
LDL-cholesterol# (mg/dL)	-0.0035	0.0006	2.46×10^{-8}
Triglyceride# (mg/dL)	0.0015	0.0012	0.1956
Glucose metabolism			
Fasting plasma glucose†† (mg/dL)	0.1086	0.0751	0.1479
HbA1C†† (%)	0.0003	0.0030	0.9178
Uric acid			
Uric acid††† (mg/dL)	-0.0018	0.0055	0.7408
Renal function			
Creatinine (mg/dL)	0.0005	0.0013	0.6823
eGFR (mL/min/1.73 m^2)	0.0852	0.1074	0.4273
Albuminuria (mg/L)	-0.0003	0.0023	0.8799
Liver function			
AST (U/L)	-0.0103	0.0599	0.8640
ALT (U/L)	-0.0487	0.0926	0.5988
γGT (U/L)	0.1072	0.1549	0.4891
Serum albumin (g/dL)	-0.0011	0.0011	0.3415
Total bilirubin (mg/dL)	-0.0026	0.0013	0.0488
Hematological parameters			
Leukocyte count (10^3/μL)	0.0133	0.0076	0.0811
Hematocrit (%)	-0.0313	0.0173	0.0702
Platelet count (10^3/μL)	0.6817	0.2825	0.0158
Red blood cell count (10^6/μL)	-0.0089	0.0022	4.37×10^{-5}
Hemoglobin (g/dL)	-0.0080	0.0061	0.1921
Atherosclerotic risk factors			
Diabetes mellitus (%)	0.0125	0.0178	0.4828
Hypertension (%)	0.0035	0.0132	0.7923
Current smoking (%)	0.0068	0.0181	0.7088
Gout (%)	0.0174	0.0265	0.5121
Microalbuminuria (%)	-0.0173	0.0159	0.2768
Metabolic syndrome (%)	-0.0201	0.0137	0.1441

Abbreviations, adjusted condition and participant enrollment as in Supplementary Table S1.

Supplementary Table S8. Stepwise linear regression analysis for serum albumin levels: including *GCKR* and *APOE* region polymorphisms

	serum albumin (g/dL)		
	beta	r^2	*p* value
Sex (male *vs*. female)	-0.1179	0.0501	$<10^{-307}$
Age (years)	-0.0034	0.0242	$<10^{-307}$
Body mass index (kg/m^2)	-0.0033	0.0027	1.41×10^{-51}
Current smoking (%)	-0.0205	0.0006	1.35×10^{-12}
rs1260326 (TT vs. TC vs. CC)	0.0206	0.0032	1.73×10^{-73}
rs143881585 (GG vs. GA vs. AA)	0.0346	0.0005	1.93×10^{-11}
rs146175795 (GG vs. GA)	0.0410	0.0004	3.28×10^{-9}
rs8179206 (AA vs. AG vs. GG)	0.0194	0.0004	1.63×10^{-8}
APOE rs7412 (CC vs. CT vs. TT)	--	--	--
APOC1 rs438811 (CC vs. CT vs. TT)	-0.0068	0.0002	3.80×10^{-6}
APOE- APOC1 rs439401 (TT vs.TC vs. CC)	--	--	--
